# Antiviral activity of *Micrococcus luteus* against the infection of *bean yellow mosaic virus* in faba bean

**DOI:** 10.3389/fpls.2025.1741491

**Published:** 2026-01-27

**Authors:** Mohsen Mohamed Elsharkawy, Faisal Ay Alzahrani

**Affiliations:** 1 Department of Agricultural Botany, Faculty of Agriculture, Kafrelsheikh University, Kafr Elsheikh, Egypt; 2 Department of Chemistry, College of Sciences and Arts, King Abdulaziz University, Rabigh, Saudi Arabia

**Keywords:** bean yellow mosaic virus, faba bean, gene expression, induced resistance, *Micrococcus luteus*, qRT-PCR

## Abstract

**Introduction:**

Plant viruses severely affect agricultural crops and are the cause of almost half of all major plant diseases. No successful antiviral agents are now widely available for agricultural use against phytoviruses.

**Methods:**

*Micrococcus luteus* was collected from the rhizosphere of faba bean and molecularly characterized via the 16S rRNA (Acc# PV650302). Soil inoculation greatly enhanced growth and induced systemic resistance to BYMV (Bean yellow mosaic virus) infection in faba bean plants grown in the greenhouse or field conditions.

**Results and Discussion:**

Soil drenching application of Micrococcus luteus resulted in a 78% decrease in the severity of the disease and a 70% decrease in viral accumulation levels. Superoxide dismutase (SOD), total chlorophyll content, antioxidant enzymes like catalase (CAT), ascorbate peroxidase (APX), and polyphenol oxidase (PPO) were all significantly increased after *M. luteus* treatment. The levels of oxidative stress indicators, such as malondialdehyde (MDA) and hydrogen peroxide (H_2_O_2_), were shown to be much lower after *M. luteus* treatment. The transcripts of genes involved in pathogenesis were found to be upregulated with these alterations. It is possible to use *M. luteus* as a biocontrol agent, which is a practical and environmentally friendly way to protect faba bean plants against BYMV infection, since it may increase faba bean growth and generate systemic resistance against BYMV disease. Antiviral action against viral infections in plants has never been previously documented for *M. luteus*.

## 1 Introduction

Plant diseases, particularly plant viruses, are a major worldwide issue that jeopardize food security via crop losses (Elsharkawy et al., 2012). The quality and yield of agricultural crops are seriously affected when plants get infected with viruses (Aseel et al., 2023). The wide host range and many transmission means of plant viruses make them very difficult to manage in different types of crops (Elsharkawy et al., 2012). The faba bean’s vulnerability to both biotic and abiotic stressors have resulted in yield losses. As one of the most major plant viruses, BYMV (single-stranded RNA) belongs to the Potyvirus family and has been detected in more than 60 distinct plant species (Chatzivassiliou, 2021). BYMV has a considerable economic effect because of its spread via several mechanisms, including mechanical inoculation, contaminated seeds, and aphids (Elsharkawy et al., 2019). BYMV infection severely affects the economically important faba bean crop, resulting in decreased agricultural productivity (Chatzivassiliou, 2021). Crop losses of 30% or more may occur as a consequence of yellowing, mottling, and necrosis brought on by the BYMV infection, which also reduces the area of the leaves and the number of flowers produced (Jha et al., 2023). The decline of faba bean shoots and roots after BYMV infection has also been associated with crop losses, according to previous research (Elhelaly, 2022).

Two significant issues that seriously affect the continuous supply of food are the extensive use of harmful pesticides and agricultural practices and chemicals for nutritional products. The extensive use of chemical pesticides has negative effects on both human and crop health due to the pollution and toxic waste they release (Saeed et al., 2021). The use of agrochemicals to combat plant viruses has had negative consequences for the environment, food product safety, farmers, and the appearance of resistance to pesticides, as well as for the eradication of beneficial and non-target microbes (Piccoli et al., 2016). The development of new resistant plants and efforts to reduce the viral transmission vector are two examples of the more realistic approaches that have recently been suggested. Although developing resistant cultivars via genetic engineering is crucial for plant disease prevention, there is still a chance that the infectious agent will find a way to defeat this protection. A more effective and eco-friendly way to manage plant viruses is to increase plant immune systems. In a wide range of environments, PGPR (plant growth-promoting rhizobacteria) have been shown to enhance plant development (Elsharkawy et al., 2023). Plants treated with PGPR show improved growth in response to a wide range of biotic and abiotic stresses (Rizvi et al., 2022). Through increasing nutrient absorption or blocking the infectious agent, PGPR has been shown in several studies to boost plant growth and increase resistance to viral infections (Wang et al., 2021). A sustainable and effective technique for treating plant viral diseases is to enhance their systemic immunity (Elsharkawy et al., 2012; Abdelkhalek et al., 2022). Inducing systemic resistance and improving plant development, PGPRs have the ability to produce bioactive compounds that activate several plant defense genes against viruses and/or enhance nutrient absorption (Abdelkhalek et al., 2020; Elsharkawy et al., 2022). The longevity and adaptation of the PGPR inoculum to the local microbiota are strain-dependent and greatly influenced by application approaches, which often provide conflicting outcomes when tested in the field (Pellegrini et al., 2020).

The main objective of studying *Micrococcus luteus* was to identify its potential to stimulate plant growth (Raza and Faisal, 2013; Shahzad et al., 2017). In nature, plants face both biotic and abiotic stresses simultaneously, which reduces crop yields (Ramegowda and Senthil-Kumar, 2015). However, research on PGPRs that can withstand both types of stress is limited. *M. luteus* can be used to promote growth of soybean and provide protection against *Fusarium oxysporum* (Dubey et al., 2021). Therefore, potential application approaches of PGPR might be used (El-Gendi et al., 2022). This research aimed to determine whether *M. luteus* may improve faba bean growth and resistance to BYMV infection by different application methods.

## 2 Materials and methods

### 2.1 Rhizobacterial isolation and identification

Fifteen soil samples collected from the rhizosphere of faba bean fields in Kafr Elsheikh (Kafrelsheikh University), Egypt, were the original source of the bacteria. The most potent antiviral efficacy was the determining criterion in selecting the optimum isolate. After 48 h of incubation at 30°C, the colonies were properly purified and inoculated on new nutrient agar plates to guarantee complete separation and remove any possible background bacteria. A streaking approach was applied at least five times. On nutritional agar slants, thirty distinct strains of pure bacteria were cultivated. Physiological and morphological characteristics from Bergey’s Manuals were used in conjunction with universal primers for 16S ribosomal DNA to identify isolates (Elsharkawy et al., 2022). Phylogenetic tree, constructed using the MEGA12 program, was used to display the evolutionary relationship, and the annotated sequences have been recorded in the Gen-Bank database. The BYMV isolate, which has been identified and characterized in our previous study, was used in this study (Elkhyat, 2018; Elsharkawy et al., 2019).

### 2.2 Greenhouse experiment

Insect-proof greenhouse pot experiments were conducted to evaluate the effectiveness of *M. luteus* in increasing the systemic resistance of faba beans to BYMV infection. The Egyptian Agriculture Research Center provided the virus-free seeds for faba bean culativar Giza 843. After sterilizing the seeds (ethanol followed by thorough rinsing with sterile water), they were placed in plastic pots (25-cm) with a sterilized combination of peat moss, sand, and clay in the ratio of 1:1:1. The plants were chosen based on their identical length and then split into four equal groups, with ten plants per each group. Initial treatment consisted of healthy plants serving as a negative control (NC). Plants infected with BYMV were classified as part of the second group (PC). Two more treatments included seed coating (SC) and soil drenching (SD) with *M. luteus* (1×10^8^ CFU/mL) were used following the method provided by Elsharkawy and El-Sawy (2019). Three independent technical duplicates were used to assess each biological replicate.

### 2.3 BYMV inoculation

Mechanical inoculation method was used to infect the top two leaves (three weeks old plants) with BYMV and potassium phosphate buffer, pH 7.0 (Elsharkawy et al., 2019). Daily symptoms development monitoring was conducted on all plants cultivated in the insect-proof greenhouses for one month. Severity of BYMV (DS) was assessed numerically based on a scale from 0 to 3 (Abdelkhalek et al., 2025). On this scale, 0 indicates no symptoms, 1 mild mosaic, 2 severe mosaic, and 3 malformation. The following equation was utilized to determine the DS values and expressed as a percentage.


$$\text{DS}=\frac{\sum (Disease\;scale\;\times \;Number\;of\;plants\;per\;scale)}{\left(Total\;number\;of\;plants\;\times \;The\;highest\;disease\;scale\right)}\;x\;100$$

The titer of virus in faba bean leaves was measured 10-, 20-, and 30-days post BYMV infection (DPI) using the Indirect Enzyme-Linked Immunosorbent Assay (ELISA) method (Elsharkawy et al., 2019). The ELISA test was conducted three times, with ten samples in each group.

### 2.4 Free radicals and oxidative stress measures

Plants’ antioxidant and reactive oxygen species scavenging capabilities were evaluated using the 2,2-diphenyl-1-picrylhydrazyl (DPPH) test. The reduction in DPPH color after mixing leaf extract (100 µL) with DPPH solution (2 mL, 0.05 M in methanol) was used to measure the free radical-quenching activity (Abdelkhalek et al., 2025). In each group, two biomarkers—lipid peroxidation and hydrogen peroxide (H_2_O_2_) accumulation—were assessed to clarify the oxidative stress levels. The accumulation of H_2_O_2_ was assessed using the modified potassium iodide technique (Abdelkhalek et al., 2025). The thiobarbituric acid (TBA) technique was also used to assess the MDA level technique (Abdelkhalek et al., 2025). The experiments were repeated three times at 20 DPI.

### 2.5 Evaluation of antioxidant enzymes

The colorimetric approach was used to evaluate the POX (peroxidase) activity in each group (Nakano and Asada, 1981). After starting the chemical reaction with 10 µL of H_2_O_2_ (10 mM), the reaction color was recorded at 290 nm. One unit of POX activity is represented by a 0.1 variation in the reaction absorbance under test conditions.

SOD (superoxide-dismutase) activity was assessed by analyzing the photoreduction of NBT (nitroblue tetrazolium) salt. It is denoted as µM/g of fresh weight. Reaction controls were generated using 100 µL of phosphate buffer.

The efficacy of the CAT (catalase) activity was assessed using the H_2_O_2_ degradation ability and measured at 240nm (Cakmak and Marschner, 1992). Under the same reaction circumstances, one unit of CAT activity is defined as the degradation of H_2_O_2_.

The method for measuring PPO (polyphenol oxidase) activity was the quinone oxidation technique (Cho and Ahn, 1999). The increase in reaction absorbance after 30 minutes at 420 nm was used to determine the PPO activity (µM/g of F.W.). The experiments were repeated three times at 20 DPI.

### 2.6 Assessment of relative expression of faba bean defense genes

The qRT-PCR method was used to investigate the way various polyphenolic and PR genes were expressed in relation to each other at 4 DPI. A total of four genes were tested, including *HCT* (shikimate hydroxycinnamoyl transferase), and *C_3_H* (Cysteine-3 Histidine, polyphenolic genes) and *PR-1* (pathogenesis related gene-1) and *PR-5* (pathogenesis related gene-5). Plant leaves were used as a source of RNA in the RNeasy plant mini kit (QIAGEN, Germany), which was used to get the total RNA from each group. RNA-DNase treatment (2 µg) was combined with the Super-Script II enzyme to generate cDNA. RNA extraction and DNase treatment were conducted in accordance with the methodology outlined by Elsharkawy et al. (2022). Actin (reference gene, Faba bean-actin) was used to normalize the transcription levels (**Table 1**). The qRT-PCR experiment was conducted in accordance with the guidelines supplied by the SYBR Green PCR Master Mix manufacturer. The target gene’s relative transcript level was properly measured and quantified using the technique of Livak and Schmittgen (2001). The experiments were repeated three times.

**Table 1:** The nucleotide sequences of the primers utilized in this study.

Primer	Forward	Reverse
*PR-1*	GTTCCTCCTTGCCACCTTC	TATGCACCCCCAGCATAGTT
*PR-5*	AATTGCAATTTTAATGGTGC	TAGCAGACCGTTTAAGATGC
*HCT*	TCT CCA ACC CCT TTT AAC GAACC	CAA CTT GTC CTT CTA CCA CAG GGA A
*C_3_H*	TTG GTG GCT ACG ACA TTC CTA AGG	GGT CTG AAC TCC AAT GGG TTA TTC C
*Actin*	GTTAGCAACTGGGATGACAT	GTTACGACCACTAGCATA GAGTG

### 2.7 Field experiment

Faba bean seeds were planted in rows (30 cm apart) in hills (20 cm apart). The seed rate was 2 seeds per hill. Other cultural practices were implemented in accordance with the faba bean’s recommendation, with four repetitions (Elsharkawy et al., 2019). Regarding pest management and fertilization, the recommended measures were implemented. A number of growth indicators, such as the plant height, leaf area and fresh and dry weights as well as yield measures in all groups of faba bean plants were measured. Furthermore, the chlorophyll meter (SPAD-502 Plus) was used to directly assess the amount of chlorophyll in plant leaves (30 DPI). The procedures used and treatment groups in the field investigation were identical to those used in greenhouse experiments.

### 2.8 Statistical analysis

Analysis of (ANOVA) variance was used to examine the effects of *M. luteus* therapy on BYMV inhibition. Experiment replication for three times verified all findings. Every statistical analysis was carried out using EKUSERU-TOUKEI (Social Survey Research Information Co., Ltd.) with a significance level of *P < 0.05*.

## 3 Results

### 3.1 Identification of the rhizobacterial strain

**Figure 1:**
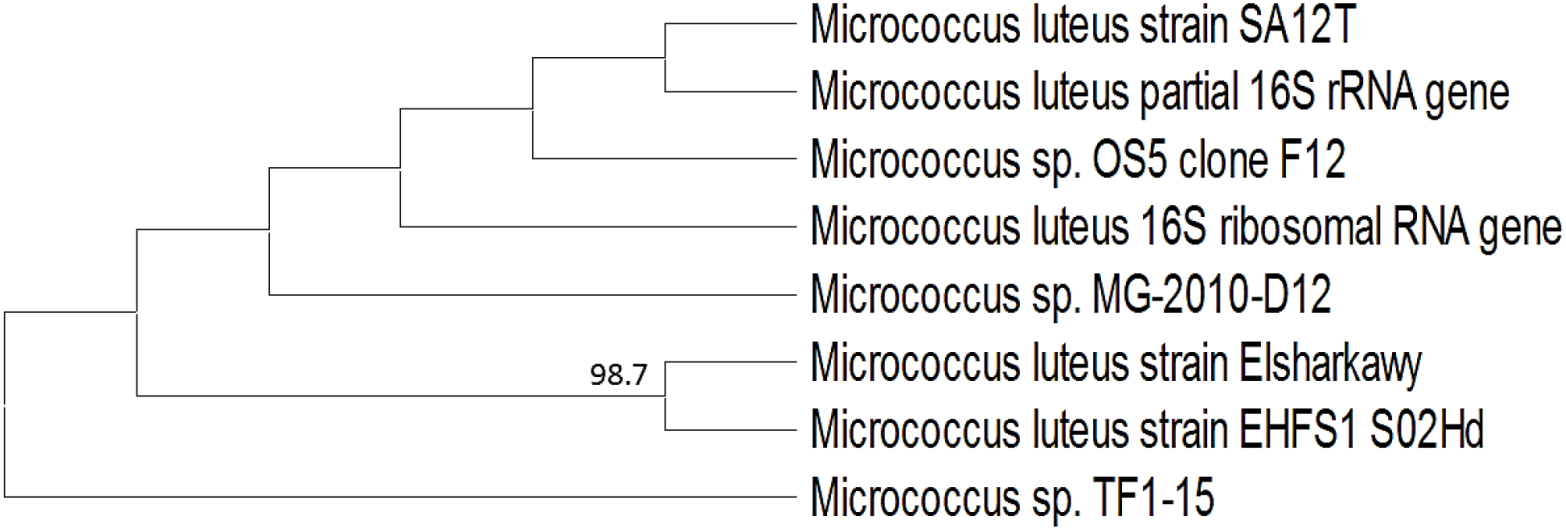
A phylogenetic tree demonstrated the connection between the identified *M. luteus* and other *Micrococcus* isolates found in GenBank.

Disease severity and mosaic symptoms on faba bean plants were used to identify the most effective bacterial isolate. One isolate was chosen for identification and future use because it exhibited the highest antiviral activity out of 30 isolates. Molecular analysis using GenBank sequence databases showed a 98.7% homology match to *M. luteus* strain EHFS1, indicating a close relationship between the two species. *M. luteus* strain Elsharkawy with the accession number PV650302 has its 16S rRNA sequence deposited in GenBank. The phylogenetic tree was constructed (**Figure 1**) that shows that *M. luteus* strain Elsharkawy is closely related to other strains of *M. luteus*, particularly strain EHFS1.

### 3.2 BYMV disease severity and titer by ELISA

**Figure 2:**
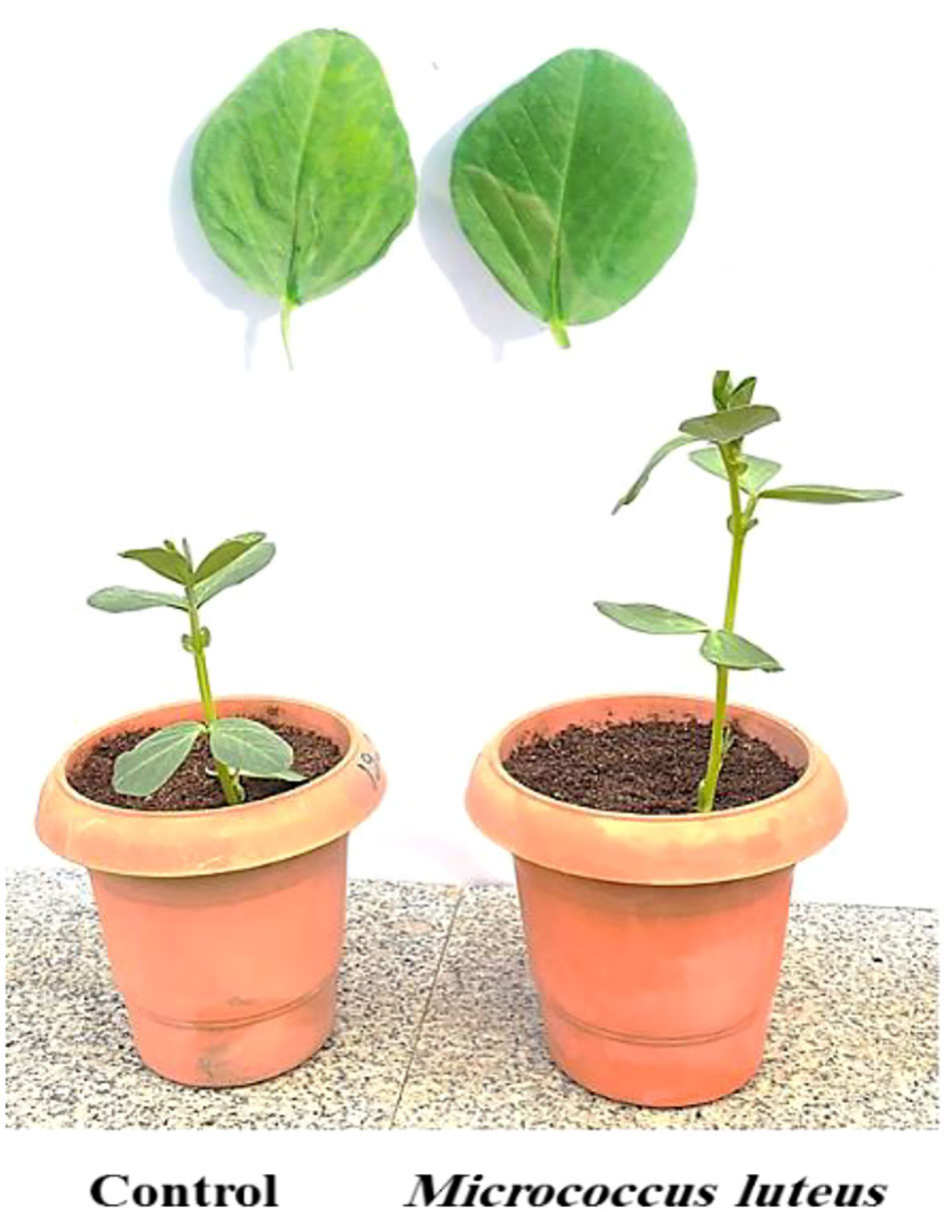
Faba bean leaf disease symptom development after 10 days post-inoculation with BYMV. Control group refers to *Bean yellow mosaic virus* infected plants and the *M. luteus* group received soil drenching treatment.

**Figure 3:**
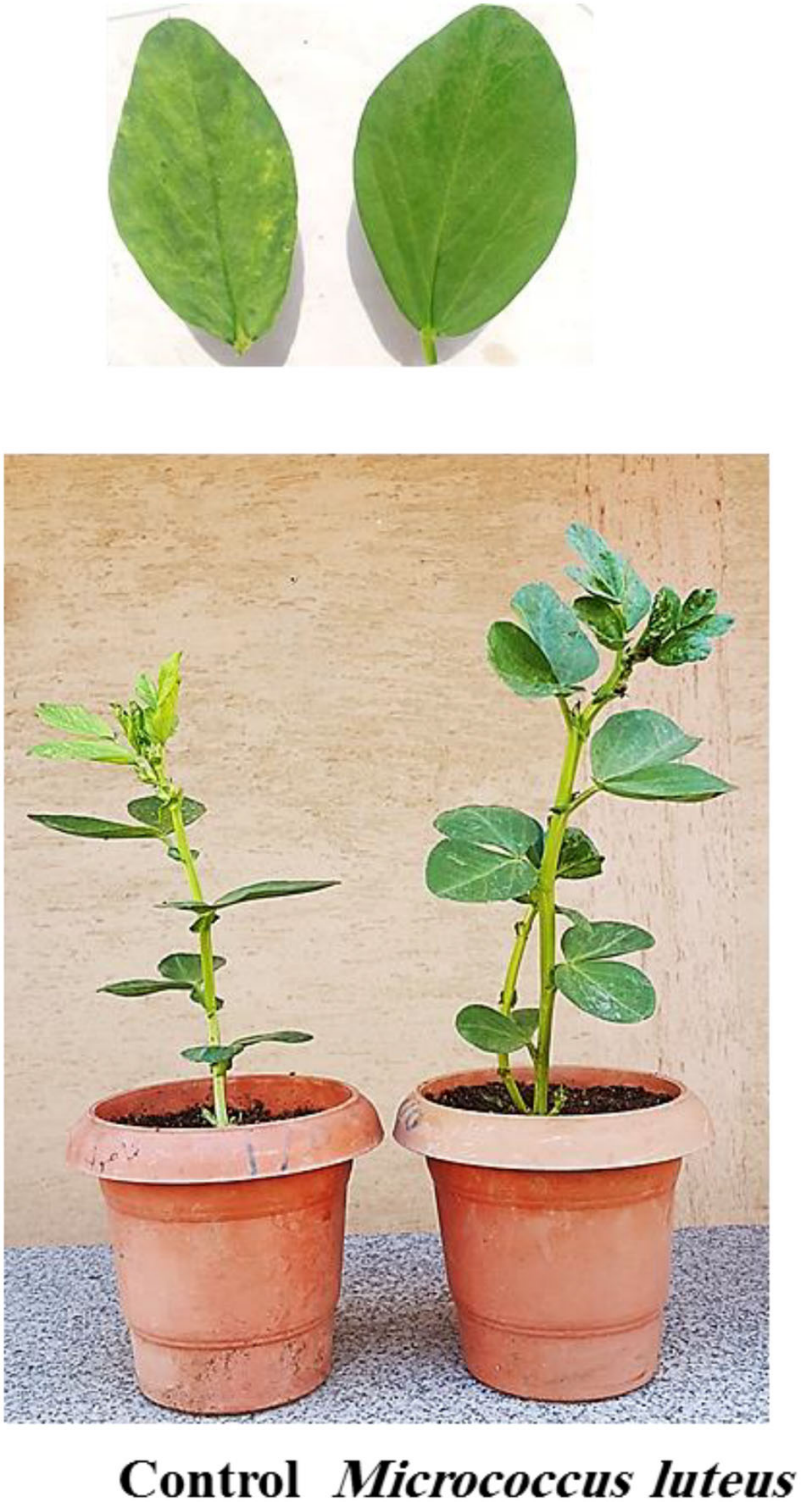
Faba bean leaf disease symptom development after 20 days post-inoculation with BYMV. Control group refers to *Bean yellow mosaic virus* infected plants and the *M. luteus* group received soil drenching treatment.

**Figure 4:**
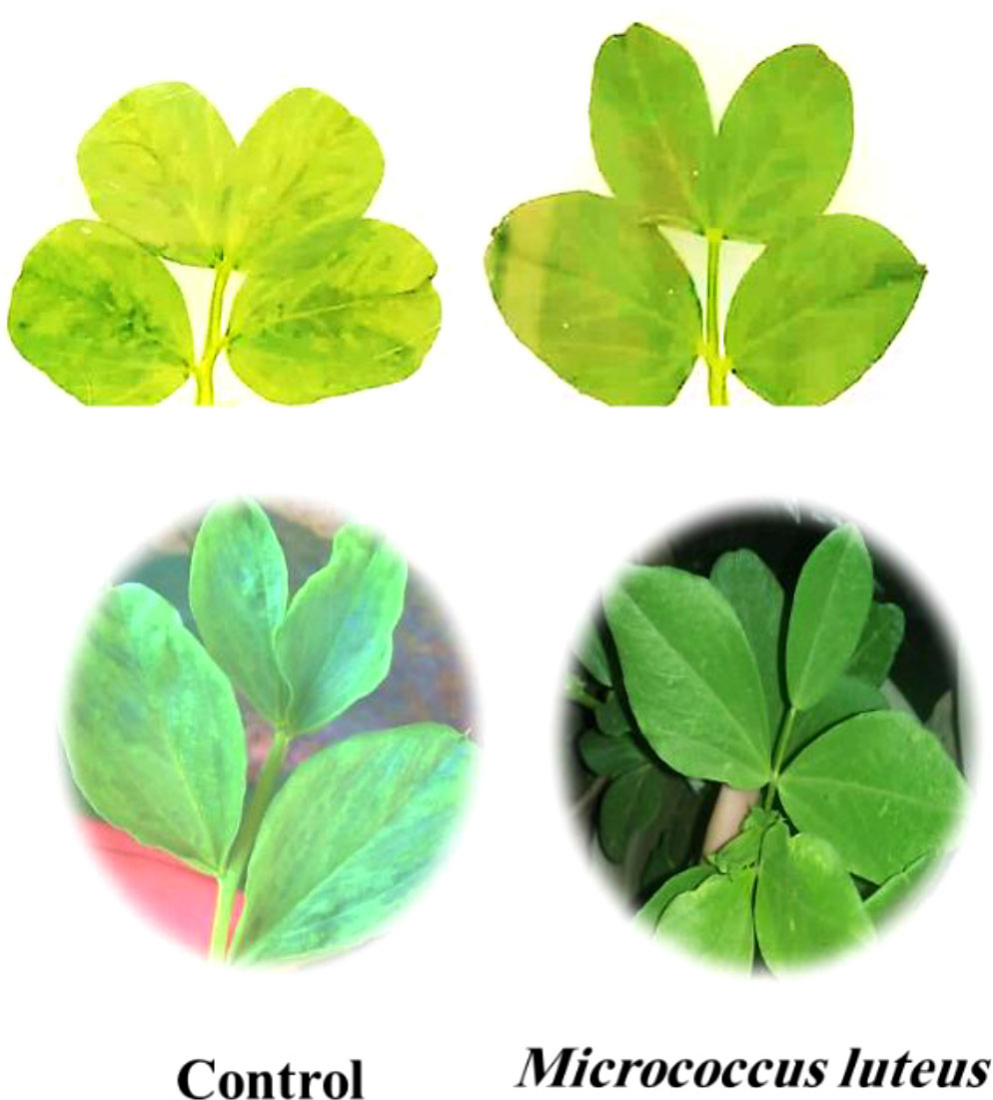
Faba bean leaf disease symptom development after 30 days post-inoculation with BYMV. Control group refers to *Bean yellow mosaic virus* infected plants and the *M. luteus* group received soil drenching treatment with 2x magnification.

**Figure 5:**
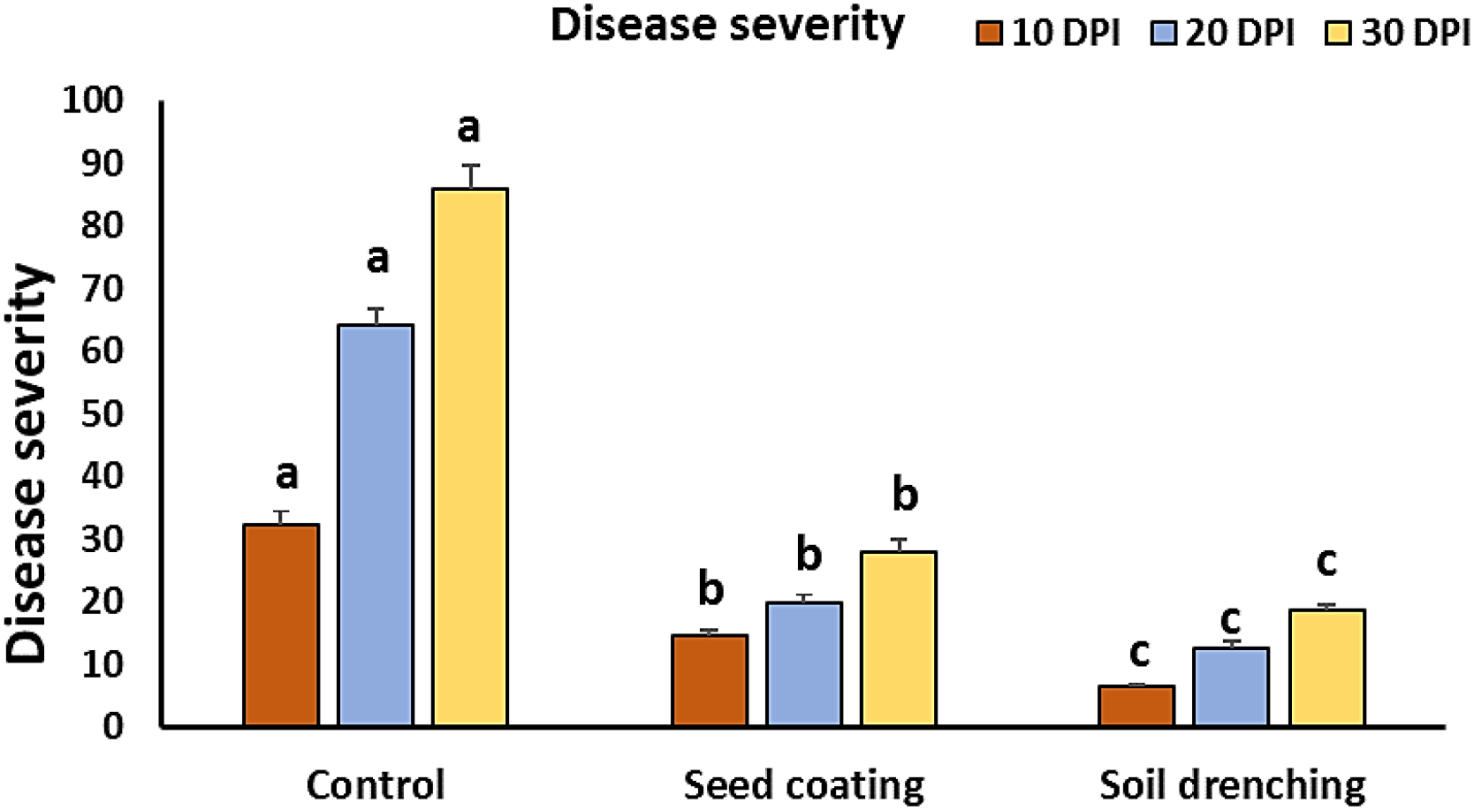
Assessment of faba bean plant disease severity following *Bean yellow mosaic virus* challenge at 10, 20- and 30-days post-inoculation. Control group refers to BYMV infected plants and the *M. luteus* group received seed coating and soil drenching treatments. Different letters denote statistically significant differences among treatments.

**Figure 6:**
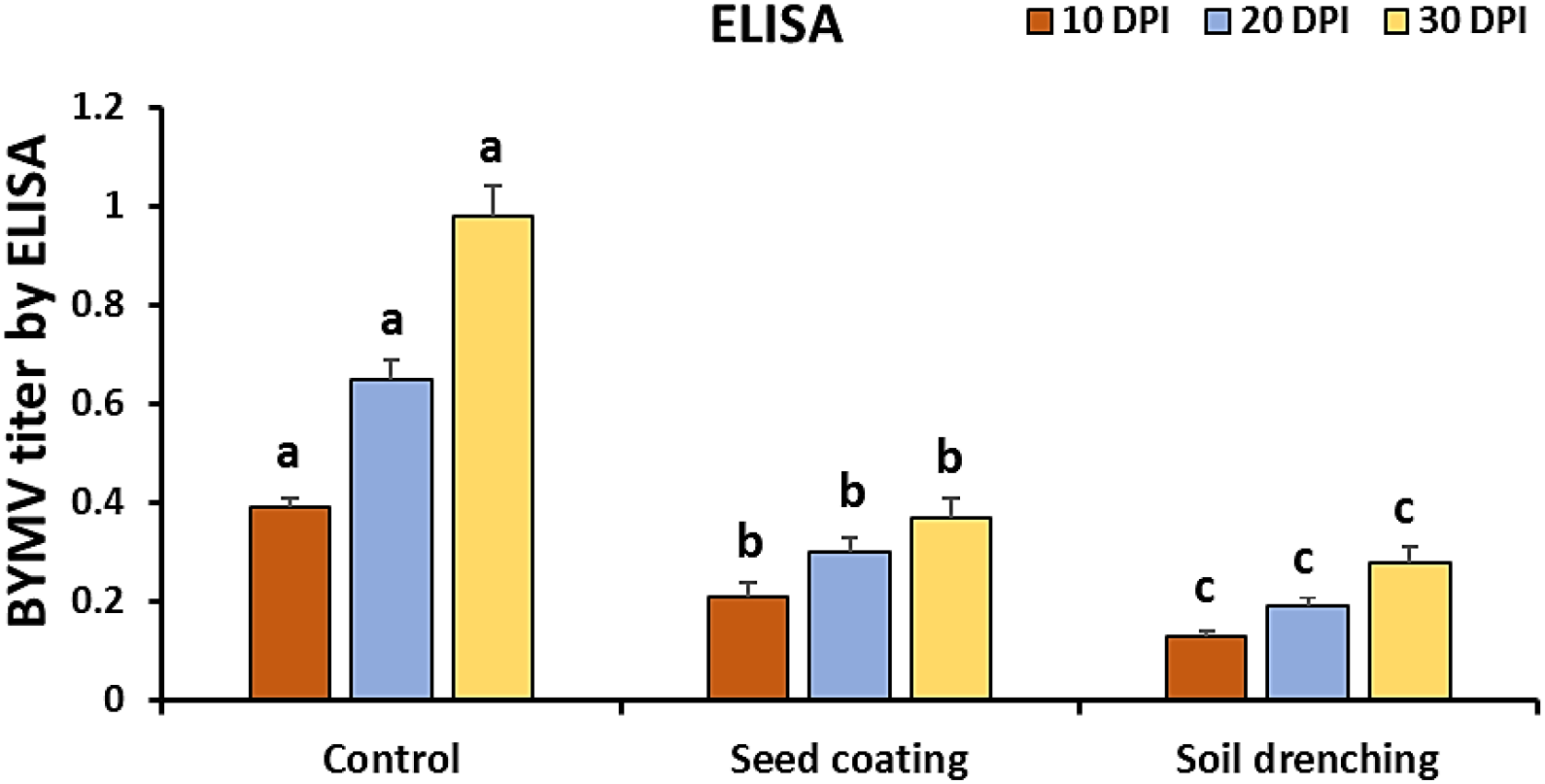
Estimation of BYMV titer following *Bean yellow mosaic virus* challenge at 10, 20- and 30-days post-inoculation. Control group refers to BYMV infected plants and the *M. luteus* group received seed coating and soil drenching treatments. Different letters denote statistically significant differences among treatments.

The BYMV-inoculated plants began exhibiting mild mosaic symptoms at 10 days post-inoculation in the greenhouse (**Figure 2**). Visible mosaic symptom started at 20 DPI, while severe mosaic symptoms culminated in deformity at 30 DPI (**Figures 3**, **4**). It is noteworthy that symptoms appeared around 7 days later when *M. luteus* was treated to the soil (soil drenching) or the seeds (seed coating treatment), with minor symptoms appearing at 20 DPI (**Figure 3**). The BYMV treatment reached up to 85.9% disease severity (DS) (**Figure 5**). These outcomes are consistent with the BYMV accumulation levels by ELISA (**Figure 6**). The soil drenching treatment of *M. luteus* demonstrated an effective method of application for BYMV titer and disease severity to 18.5%, and 0.28, respectively at 30 DPI. However, the seed coating of *M. luteus* group reported a 27.9% disease severity, and a 0.37 viral accumulation (**Figures 5**, **6**).

### 3.3 Free radicals scavenging activity and oxidative stress markers

According to the findings (**Table 2**), the BYMV group’s free radical scavenging activity was marginally higher than the healthy group. *M. luteus* treatments increased free radical scavenging to 91.7% and 98.6% with seed coating (SC) and soil drenching (SD) applications, respectively. The BYMV group contained 2 times as many H_2_O_2_ molecules as the healthy plants (**Table 2**). When compared to the BYMV group, the treatment groups (SC and SD) showed significant decreases in H_2_O_2_ buildup to 6.9 and 6.5, respectively. Similarly, as shown in **Table 2**, the BYMV group’s MDA level was significantly higher than that of the healthy plants. *M. luteus* treatment reduced the MDA level in SC and SD to 84.6 and 82.9 µM/g F.W., respectively.

**Table 2:** Effect of *M. luteus* treatments on total oxidative state using the 2,2-diphenyl-1-picrylhydrazyl (DPPH) assay and hydrogen peroxide (H_2_O_2_) of faba bean plants.

Treatments	H_2_O_2_ (µM/g F.W)	DPPH (%)	MDA (µM/g F.W)
Healthy	4.6 c	81.6 d	77.3 c
Infected	9.7 a	85.9 c	103.6 a
Seed coating	6.9 b	91.7 b	84.6 b
Soil drenching	6.5 b	98.6 a	82.9 b

Infected group refers to *Bean yellow mosaic virus* infected plants and the *M. luteus* group received seed coating and soil drenching treatments. Different letters (a, b, c, d) denote statistically significant differences among treatments.

The findings showed that the BYMV treatment resulted in higher SOD (superoxide dismutase level) (0.52 µM/g F.W.) as compared to the healthy plants (0.44 µM/g F.W.). The SC and SD groups had significantly greater levels of SOD, at 0.63 and 0.79 µM/g F.W., respectively. In comparison to the healthy plants (0.35 µM/g F.W.), the BYMV group had greater CAT activation (0.47 µM/g F.W.) (**Table 3**). In the SC group, CAT expression was increased to 0.60 µM/g F.W., whereas in the SD group, it was increased to 0.58 µM/g F.W. by treatment with *M. luteus*.

**Table 3:** Effect of *M. luteus* treatments on antioxidant enzymes activity of faba bean plants.

Treatments	POX (µM/g F.W)	PPO (µM/g F.W)	SOD (µM/g F.W)	CAT (µM/g F.W)
Healthy	0.14 d	0.34 d	0.44 d	0.35 c
Infected	0.19 c	0.49 c	0.52 c	0.47 b
Seed coating	0.24 b	0.57 b	0.63 b	0.60 a
Soil drenching	0.31 a	0.69 a	0.79 a	0.58 a

Infected group refers to *Bean yellow mosaic virus* infected plants and the *M. luteus* group received seed coating and soil drenching treatments. Different letters (a, b, c, d) denote statistically significant differences among treatments.

In regards to the POX level (**Table 3**), the activity was increased in plants infected with BYMV (0.19 µM/g F.W.) as compared to the control plants (0.14 µM/g F.W.). POX levels in the SC group increased to 0.24 and in the SD group to 0.31 µM/g F.W. According to the findings of the PPO estimates, when comparing the SC and SD groups to the infected plants, there were small increases in PPO levels to 0.57 and 0.69 µM/g F.W., respectively (**Table 3**).

### 3.4 Relative expression levels of defense genes

**Figure 7:**
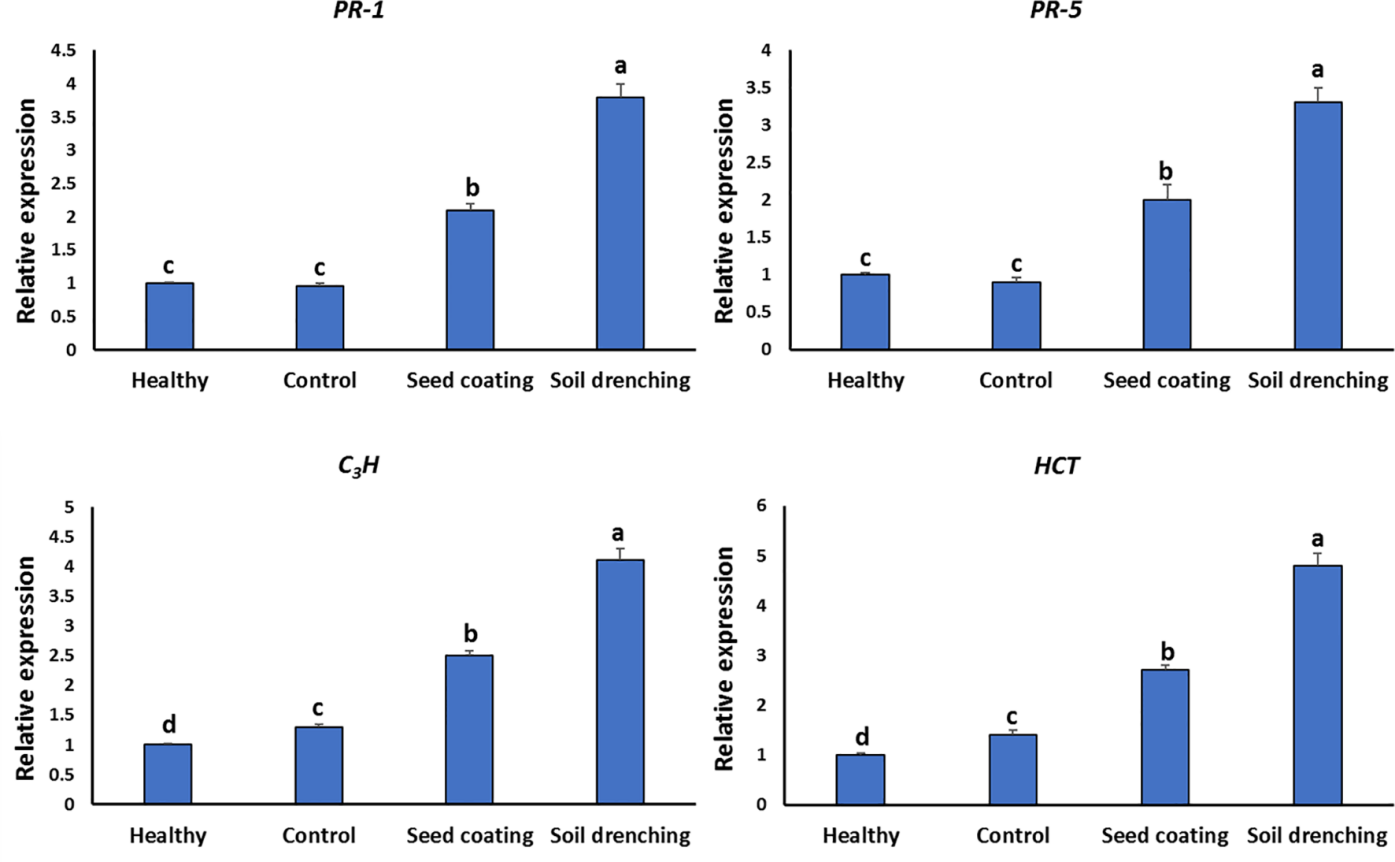
Relative expression of pathogenesis-related genes (*PR-1* and *PR-5*) and polyphenolic genes (C3H, and HCT) of healthy, *Bean yellow mosaic virus*-infected, seed coating with *M. luteus*, and soil drenching with *M. luteus*. Different letters denote statistically significant differences among treatments.

The SD group showed a significant rise in the relative accumulation of the *PR-1* gene compared to the other treatment groups (3.8 folds). The SC group showed *PR-1* expression of about 2.1 folds compared with the control (**Figure 7**). The SC and SD treatments had a maximal relative accumulation of *PR-5* that was about 2 and 3.3 times higher, respectively. In comparison to the control group, the SC and SD groups had a relative accumulation of *C_3_H* that was about 2.5 and 4.1 folds higher, respectively (**Figure 7**). In addition, the *HCT* levels in the BYMV group significantly increased with a 1.4-fold increase compared to control (**Figure 7**). The SD application achieved the highest expression levels, 4.8 folds rise. The treated group with SC showed 2.8 folds increase in *HCT* relative expression (**Figure 7**).

### 3.5 Measuring faba bean growth

A comparison with the healthy group (52.6 cm, 469 cm^2^ plant^-1^) reveals that the faba bean height and leaf area were adversely affected by the BYMV infection (40.1 cm, 253 cm^2^ plant^-1^, respectively), as shown in (**Table 4**). Both fresh and dry weights in the BYMV group significantly decreased to 62.9 and 5.1 g, respectively, as compared to the healthy group 115.9 and 10.7 g, respectively, due to the shortening of the plant length. Faba bean growth was improved by the *M. luteus* treatments in response to BYMV infection. Plant development and the process of photosynthesis are directly indicated by the amount of chlorophyll available. The direct effect of BYMV on chlorophyll content is shown (**Table 4**), BYMV infection significantly decreased the chlorophyll content to 0.51 uE in comparison to the healthy group (0.77 uE). This decrease was significantly improved to 0.70 and 0.75 uE by treatments with *M. luteus* using both SC and SD methods, respectively. The SD treatment of *M. luteus* resulted in better growth increase in terms of plant height, leaf area, chlorophyll contents and fresh and dry weights (49.5 cm, 419 cm^2^ plant^-1^, 0.75 uE, 101.2 g and 9.5 g, respectively).

**Table 4:** Effect of *M. luteus* treatments on faba bean growth indices at 6 weeks after planting.

Treatments	Plant height (cm)	Leaf area (cm^2^ plant^-1^)	Chlorophyll content (uE)	Fresh weight (g plant^-1^)	Dry weight (g plant^-1^)
Healthy	52.6 a	469 a	0.77 a	115.9 d	10.7
Infected	40.1 d	253 d	0.51 c	62.9 c	5.1
Seed coating	46.5 c	369 c	0.70 b	90.6 b	7.3
Soil drenching	49.5 b	419 b	0.75 a	101.2 a	9.5

Infected group refers to *Bean yellow mosaic virus* infected plants and the *M. luteus* group received seed coating and soil drenching treatments. Different letters (a, b, c, d) denote statistically significant differences among treatments.

There was a statistically significant drop in seed yield per plant when faba bean plants were infected with BYMV, as shown in (**Table 5**). In addition, there were no significant variations in the quantity of pods produced per plant between healthy plants and plants treated with SD of *M. luteus* and infected with BYMV (**Table 5**). Additionally, as compared to the control treatment, plants that were treated with SC and SD produced more seeds and yield per plant.

**Table 5:** Effect of seed coating and soil drenching treatments of *M. luteus* on faba bean yield parameters compared with bean yellow mosaic virus infected group and healthy group.

Treatments	Bods (No. plant^-1^)	Seeds (No. plant^-1^)	100-seed weight (g)	Seed yield (g. plant^-1^)
Healthy	12.9 a	37.9 a	74.5 a	28.6 a
Infected	7.3 c	14.7 d	51.9 d	9.4 d
Seed coating	11.1 b	30.5 c	63.5 c	19.4 c
Soil drenching	12.6 a	34.1 b	71.7 b	24.6 b

Different letters (a, b, c, d) denote statistically significant differences among treatments.

## 4 Discussion

In light of the difficulty and variability of managing viral infections in plants and the constant changes to their surrounding environment, the search for novel biocontrol agents is of the utmost importance (Etesami et al., 2023). Research has shown that certain strains of beneficial bacteria may really help plants by preventing harmful diseases. *Bacillus* strains were utilized extensively in the management of viral diseases including *Cucumber mosaic virus*, *Tobacco mosaic virus*, *Tomato* sp*otted wilt virus*, *Potato virus Y, Potato virus X*, and *Tomato yellow leaf curl virus* to decrease viral accumulation in plants and to exhibit improved antivirus actions (Kumar et al., 2024). The role of *M. luteus* in inducing plant defense systems in response to viral infections has not been investigated in any prior studies. The main emphasis of previous research has been to understand the way rhizobacteria could manage and reduce plant bacterial and fungal infections (Xu et al., 2018; Elsharkawy et al., 2023). This is why we explore *M. luteus*’s ability to inhibit BYMV replication in faba beans. Sequence analysis corroborated the morphological features of the rhizobacterial strain, which were in agreement with those of *M. luteus*. GenBank has received the annotated sequences and given them the entry code PV650302. Several studies have shown that rhizobacteria were effective as biocontrol agents (Gorai et al., 2021; Elsharkawy et al., 2022). *M. luteus* treatments stimulate faba bean plants to grow faster with better chlorophyll contents and yield. Soil drench application had an even stronger effect than seed coating application. This finding is consistent with previous research showing that BYMV inhibits the infected plant’s ability to grow (Abdelkhalek et al., 2022). Virus infection could lead to changes in chlorophyll levels, which might be a sign of the capacity of photosynthesis in plants (Petrova et al., 2012). The quantity of chlorophyll in this research was reduced by about 34% due to the BYMV infection. The results demonstrate that seed coating and soil drenching applications affect chlorophyll levels, disease severity and virus concentration directly. Soil drenching application of *M. luteus* have better direct impacts than seed coating treatment. The BYMV group claimed that *M. luteus* might control viral infection by preventing the virus from moving through the plant, while the *M. luteus* group demonstrated a 70% reduction in BYMV titer in soil drenching treatment and 61% reduction in seed coating treatment. Indirect effects of *M. luteus* include limiting viral replication and reducing the oxidative impact of viral infections by enhancing antioxidant enzyme activity and encouraging plant development. The results indicate that *M. luteus* could suppress BYMV infection and impact the development of systemic resistance. Researchers have shown that rhizobacterial treatment may postpone the start of viral symptoms (Elsharkawy et al., 2022). Antioxidant enzymes help plants indirectly fight against a variety of phytopathogens by reducing oxidative stress within their own cells (Khan et al., 2023). Plants treated with *M. luteus* and infected with BYMV exhibited elevated levels of SOD, CAT, APX, and PPO enzymatic activities compared to the control group. Enzymes with antioxidant characteristics are vital for preventing pathogen penetration by strengthening cell walls and reducing tissue oxidation (Elsharkawy et al., 2023). Enzyme activity was greatest in plants that were treated with *M. luteus*. The results suggest that rhizobacteria, such as *M. luteus*, use comparable approaches to control viral infections in plants. In contrast, the rising levels of H_2_O_2_ and MDA in BYMV-infected faba bean plants are consistent with the normal plant response to viral infection and the buildup of ROS (El-Gendi et al., 2022; Zhu et al., 2021). Ultimately, the catalase enzyme neutralizes H_2_O_2_, which is often thought of as part of the detoxification process of free superoxide free radicals (O^2−^) (Baker et al., 2023). An important finding is that *M. luteus* application is associated with significantly lower levels of oxidative stress indicators. Reducing the activity of oxidative stress-inducing enzymes helps keep cell membranes stable and intact (Balal et al., 2012). The results show that *M. luteus* effectively reduces oxidative stress in virus-infected plants, as shown by the significant decrease in MDA and H_2_O_2_ levels.

This work highlights the significance of *PR-1* in faba bean resistance against BYMV infection, since its relative formation value in the *M. luteus* treatments is almost four times higher than in the healthy group. The findings showed that *M. luteus* treatments, particularly when applied as soil drenching, were very effective in inducing systemic resistance-mediated plant processes such as *PR-5*, *C_3_H* and *HCT* expressions. The expression of *PR-5* is regulated by SA (Wang et al., 2021). The first enzyme involved in the manufacture of chlorogenic acid is HCT. It facilitates the conversion of p-coumaroyl CoA to shikimate. Chlorogenic acid is produced when C3H converts shikimate to p-coumaroyl shikimate (Hoffmann et al., 2003). Caffeic acid and quinic acid may be esterified to produce chlorogenic acid, a polyphenolic molecule classified as a phenolic acid. A number of publications emphasized the fact that it might increase plant disease resistance (Leiss et al., 2009). Therefore, results demonstrated that *M. luteus* altered the faba bean plant’s signaling pathways, resulting in induced systemic resistance against BYMV.

Factors such as formulation, technique, transportation, and storage conditions all impact the stability of PGPR (El-Saadony et al., 2022; Alamoudi, 2023). In order to get very high BCA survival rates, formulation should be improved. Reducing the storage temperature and/or changing the mixtures of additives are two ways that many scientists have tried to increase PGPR’s shelf life (Berger et al., 2018).

## 5 Conclusion

Plants treated with *M. luteus* as a soil drenching showed a 78% decrease in disease severity and a 70% decrease in BYMV accumulation levels at 30 DPI. The antioxidant enzyme activities, total chlorophyll content, DPPH, and shoot and root growth were all significantly enhanced in faba bean plants that were treated with *M. luteus*. Additionally, PR genes transcriptional levels increased and oxidative stress indicators were significantly reduced. The present investigation on *M. luteus*’s antiviral effectiveness against BYMV infection is the first of its field. The long-term effectiveness and consistency of *M. luteus* performance in practical agricultural circumstances will be evaluated through comprehensive, multi-year field studies that span several geographic regions and cropping systems.

## Data Availability

The raw data supporting the conclusions of this article will be made available by the authors, without undue reservation.
